# Deltamethrin resistance in Chagas disease vectors colonizing oil palm plantations: implications for vector control strategies in a public health-agriculture interface

**DOI:** 10.1186/s13071-020-04048-8

**Published:** 2020-04-03

**Authors:** Johan M. Calderón, Patricia Fuya, Liliana Santacoloma, Camila González

**Affiliations:** 1 Centro de Investigaciones en Microbiología y Parasitología Tropical (CIMPAT), Departamento de Ciencias Biológicas, Universidad de Los Andes, Bogotá D.C., Colombia; 2 Laboratorio de Entomología, Instituto Nacional de Salud, Bogotá D.C., Colombia

**Keywords:** Chagas disease, Triatominae, Insecticide resistance, Biological pest control, Oil palms

## Abstract

**Background:**

Triatomine bugs are responsible for the vectorial transmission of the parasite *Trypanosoma cruzi*, etiological agent of Chagas disease, a zoonosis affecting 10 million people and with 25 million at risk of infection. Several triatomine species of the genus *Rhodnius* have been found inhabiting palm crowns where insects can find shelter in leaves axils and blood from palm-associated vertebrates. *Rhodnius prolixus* insects have been collected in oil palms in Colombia, and high *T. cruzi* infection rates were found. Since pest control is carried out in oil palm plantations, continuous exposure to insecticides could be occurring in these triatomines. Some insecticides suggested for pest control in oil palm plantations are also recommended for triatomine control in human dwellings. In this study, our objective was to assess if triatomines inhabiting oil palms exhibit resistance to deltamethrin, an insecticide used for vector control.

**Methods:**

*Rhodnius prolixus* nymphs were sampled in oil palms located in Tauramena, Colombia. To determine deltamethrin resistance, biological and biochemical assays were carried out on fifth-instar nymphs from the F1 generation. For biological assays, pure and commercial deltamethrin were used, and in biochemical assays, activities of detoxifying enzymes related to pyrethroid resistance, such as oxidases, esterases and transferases, were quantified.

**Results:**

Deltamethrin lethal dosage 50 and 90 in *R. prolixus* from oil palms was significantly higher than in those from a susceptible colony suggesting possible deltamethrin resistance. Moreover, mortality with commercial deltamethrin was very low in insects from oil palms. In biochemical assays, the activity of evaluated detoxifying enzymes was significantly higher in *R. prolixus* from oil palms than in those from the susceptible colony.

**Conclusions:**

Possible deltamethrin resistance found in *R. prolixus* insects from oil palms could threaten traditional vector control strategies in urban settings if insecticide-resistant triatomines can migrate from oil palms plantations. In palm oil producer countries such as Colombia, the oil palm plantations are growing constantly during the last years. We suggest that pest control strategies in oil palm crops should include triatomine surveillance and toxicological monitoring, especially in zones with several Chagas disease cases.
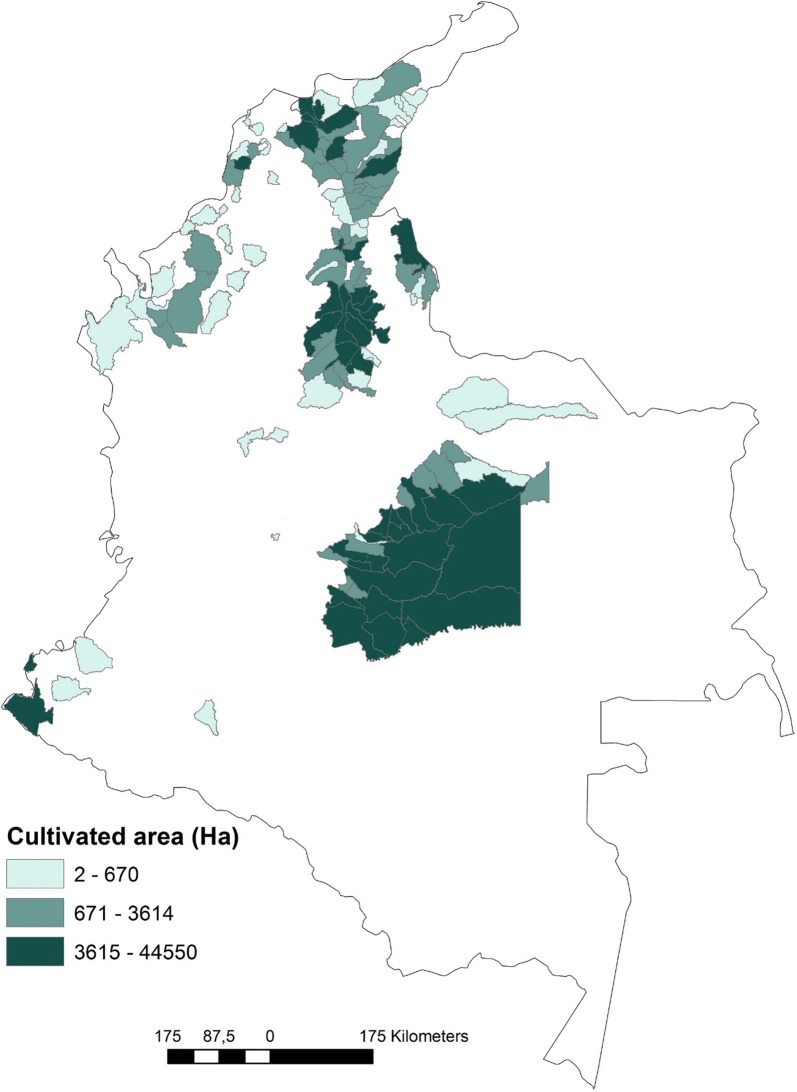

## Background

Triatomine bugs are responsible for the vectorial transmission of the parasite *Trypanosoma cruzi*, etiological agent of Chagas disease, a zoonosis affecting almost 1.3 million people in Colombia, where 3.5 million inhabit areas with high risk of infection [1]. According to the World Health Organization (WHO) [2], insecticide spraying is the main strategy for vector control in Chagas disease transmission scenarios. Chemical control is based on spraying human dwellings and peridomestic areas with insecticide formulations applied by professional sprayers [3]. This intervention has led to a reduction of the spatial distribution of important Chagas disease vectors, such as *Triatoma infestans* in the southern cone, and to interruption of parasite transmission [4].

In the 1990s, insecticide resistance in triatomines was considered unlikely due to their long life-cycle (approximately 5–6 months, but it can vary according to species) and low genetic variability [3, 5, 6]. However, in the following years, several reports of insecticide resistance in triatomines have been published. In 1966, the first evidence of triatomines surviving to insecticide dosages used in field was reported for *Rhodnius prolixus* insects from laboratory colonies exposed to organophosphates and organochlorides [7].

Three mechanisms have been found related to the development of insecticide resistance in triatomines: reduced penetration (reducing insecticide entry); enhanced metabolism (increasing insecticide degradation); and modified site of action (reducing the binding of the target with the insecticide). Reduced insecticide penetration can be the result of changes in insect cuticle [8]. Enhanced metabolism occurs when a group of detoxifying enzymes, such as monooxygenases, esterases and glutathione S-transferases, act over the insecticide reducing the amount that affects the organism [3]. A modified site of action, which is a consequence of punctual mutations [3, 6], decreases considerably insecticide-target bonding.

**Fig. 1:**
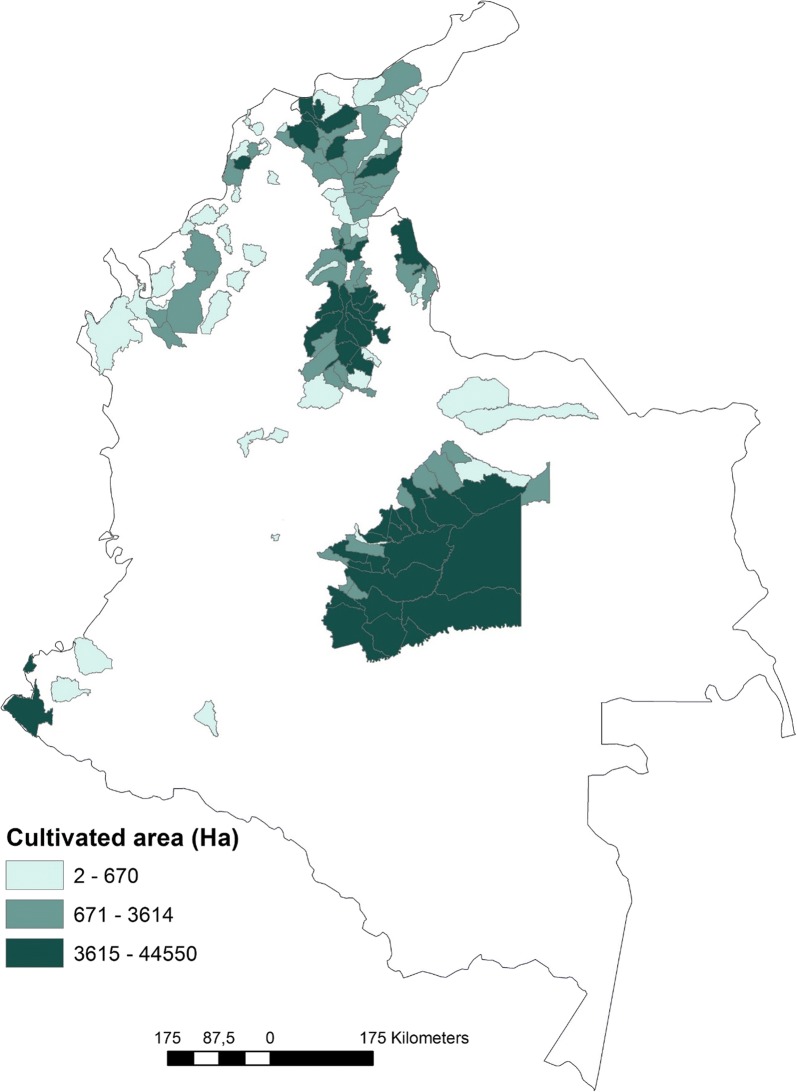
Municipalities with oil palm plantations in 2016 discriminating by cultivated area. Data source: Ministerio de Agricultura [9]. Elaborated with ArcGis 10.4.1

In the case of Chagas disease transmission, the main concern for insecticide resistance is related to the risk of vector domiciliation from palms to households, controlled with spraying. A novel epidemiological scenario has arisen in Colombia due to the introduction of massive extensions of oil palm plantations covering almost 630,000 hectares (ha) in 2016 [9]. *Rhodnius prolixus* nymphs and adults infected with *T. cruzi* have been collected from several oil palm plantations (*Elaeis guineensis* palms) [10, 11]. From 2007 to 2016, the total oil palm cultivated area in Colombia has grown by 95%, covering 127 municipalities mostly distributed in the central and north regions of the country [9] (Fig. 1). Of these, 53 municipalities have reported presence of palm-infesting triatomines (e.g. *R. pallescens*, *R. prolixus* and *Triatoma dimidiata* [12–15]) and Chagas disease cases (source: SIVIGILA); also, oil palm cultivated area in these municipalities has increased steadily, with a growth of 116% from 2007 to 2016 [9].

As any widely extended monocrop, oil palm plantations are affected by pests, which deteriorate different organs of the palm. Chemical control is as well one of the strategies used for pest control in oil palms [16, 17]. Systemic herbicides are applied in the palm stem to avoid pest colonization, and after palm pruning, exposed plant tissue is protected from pests by the application of insecticides [18]. Some insecticides suggested or used for the control of important oil palm pests are also recommended by the Pan American Health Organization (PAHO/WHO) to control triatomines in domiciles, as is the case for λ-cyhalothrin, cypermethrin and deltamethrin [19]. Alfa-cyhalothrin has been suggested to control the palm root pests *Strategus aloeus* (ox beetle) [20] and the moth *Sagalassa valida* [21, 22]; cypermethrin has been recommended to control the palm defoliator *Opsiphanes cassina* (split-banded owlet) [23], and deltamethrin has been suggested to control the important palm pest *Rhynchophorus palmarum* (South American palm weevil) [24].

Chemical pest control in oil palm plantations could be exposing triatomines living in palms to insecticides recommended for Chagas vector control. This exposure could promote the development of resistance to those insecticides in triatomines, and further, if insecticide-resistant triatomines migrate from oil palms to urban settings, traditional vector control strategies would be inefficient. Although agricultural development and public health are globally pillars of government agendas, a need for coordinated efforts may have been underestimated; as a result, important implications of independent decision-making could arise. From this perspective, the aim of this study was to assess if triatomines inhabiting oil palms exhibit resistance to insecticides used for vector control, by using biological and biochemical assays.

## Methods

### Insect sampling

Triatomine collection was carried out in Hacienda Potrillos (72° 36′ 30.2538″ W, 4° 59′ 4.311″ N) located in Tauramena (Casanare, Colombia), in an *E. guineensis* (oil palm) plantation located near (~30 m) an *Attalea butyracea* (native palm) cluster in a riparian forest. *Attalea butyracea* palms have infestation reports by *R. prolixus* in the region [25, 26]. Insect sampling was performed on July 2017 (rainy season) in 20 *E. guineensis* palms in the plantation and 20 *A. butyracea* palms in the forest. Triatomines were collected using modified Angulo traps [27], with one trap per palm from 18:00 h to 7:00 h. Traps were located in the crowns of the palms close to leaf axils. A total number of 30 *R. prolixus* nymphs were collected from *E. guineensis* and 30 *R. prolixus* nymphs from *A. butyracea*. Triatomines from native palms were collected to be used as control for sylvatic insects, assuming a lower exposure to insecticides. Collected *R. prolixus* individuals were transported alive to the Research Center for Microbiology and Tropical Parasitology (CIMPAT by its acronym in Spanish) at the Universidad de Los Andes, Bogotá, and kept in the insectary to establish a colony and obtain F1 nymphs. Insectary conditions were temperature of 26 °C and relative humidity (RH) of 76%, with 30 insects per breeding cage. Insects were fed with chicken blood for 20 min weekly. When eggs were laid, they were transferred to another breeding cage to separate F1 from parental insects.

Triatomine species identification was determined morphologically based on Lent & Wygodzinsky [28]. Additionally, species identity confirmation was carried out in 16 randomly selected individuals by sequencing a 682-bp DNA fragment of the cytochrome *b* gene using primers cytb7432F (5′-GGA CG(AT)G G(AT)AT TTA TTA TGG ATC-3′) and cytb7433R (5′-GC(AT) CCA ATT CA(AG) GTT A(AG)T AA-3′) [29].

### Biological assays

To estimate insecticide resistance, three groups of *R. prolixus* from different origins, i.e. oil palms, native palms and a reference susceptible colony (control) were tested. The control colony, maintained at CIMPAT, was established from wild populations collected in El Viso (5°00’10”N, 72°42’25”W), Mani Municipality (Casanare, Colombia) in March 2009 (no wild individuals have been further introduced into the colony).

The insecticide selected for the assays was the synthetic pyrethroid deltamethrin (DM), which is one of the insecticides recommended by the PAHO/WHO for triatomine vector control in domiciles [19]. Lethal dosages 50% (LD50) and 90% (LD90) were estimated.

Biological assays were carried out using the protocol designed by the WHO for triatomines [30]. Four DM concentrations were tested: 0.006, 0.03, 0.06 and 0.3 ng DM/insect (Deltamethrin Pestanal®, analytical standard; Sigma-Aldrich, St. Louis, USA) using acetone as solvent. For each concentration, three replicates were carried out, and for each replicate, 10 insects were used (F1 nymphs in five-instar, one week after moulting). Each insect was exposed to 0.6 µl of DM solution in the dorsal abdomen using a Hamilton® 10µl microsyringe (with a PB-600 repeating dispenser; St. Louis, USA). Nymphs were kept in Petri dishes with a 5 cm^2^ paper and transported to other insectary to protect insects in the colony (26 °C and 76% RH). Mortality was observed after 72 h. An insect was considered dead when it was placed on filter paper and there was no locomotive activity, either spontaneous or after stimulation with a paintbrush.

To estimate resistance to commercial insecticide, biological assays were performed with commercial DM formulation, K-othrine® (Bayer, Leverkusen, Germany). Filter paper disks (9 cm in diameter) were impregnated with 1 ml K-othrine® (10 ml K-othrine®/0.8 l water; this is the concentration suggested by the fabricant for the use of K-othrine® in metallic aspersers). Paper disks were air-dried for 24 h. Ten nymphs (fifth-instar) were placed in a Petri dish with an impregnated paper disk and remained there for 1 h. Then, they were moved to Petri dishes with a 5 cm^2^ paper inside and transported to the insectary (26 °C and 76% RH). Mortality was observed after 72 h. Assays were performed for insects from each origin, and a negative control (paper disks impregnated with water).

### Biochemical assays

To identify biochemical mechanisms related to insecticide resistance, enzymatic activity was evaluated in α-esterases, β-esterases, glutathione S-transferases (GST), and mixed-function oxidases (MFO) of the triatomines. These enzymes are involved in metabolic mechanisms associated to pyrethroid resistance [3]. The technique by Valle et al. [31] was used with some modifications for triatomines by Davila-Barboza et al. [32].

From each origin, 30 F1 fifth-instar nymphs were used (one week after molting). Nymphs were homogenized individually in MilliQ grade water, and the homogenate was centrifuged at 12,000× *rpm* for 60 s, after taking an aliquot for MFO assays. The homogenate was placed in wells of a flat-bottom microplate in duplicate and the absorbance was measured in a SmartReader 96 microplate reader (Benchmark Scientific, Sayreville, USA). Alpha- and β-esterase activities were determined using α-naphtyl and β-naphtyl esters as substrates (1-naphthyl acetate > 98% and 2-naphtyl acetate esterase substrate; Sigma-Aldrich) and measuring product concentration (absorbance at 570 nm). The MFO content was determined by measuring the heme content in the insect (absorbance at 650 nm). For GST, the activity was determined by conjugation of the thiol group of glutathione to the substrate, 1-chloro-2, 4-dinitrobenzene (> 99%, Sigma-Aldrich; absorbance at 340 nm). Total protein was determined by the Bradford method, which was used to correct the activity values for the evaluated enzymes.

### Data analysis

LD50 and LD90 were estimated using generalized linear models (GLM) with binomial family and probit link function (recommended by the WHO bioassays protocol). Other GLM models were tested, binomial family/logit link function and quasibinomial family, but residual deviance did not decrease (e.g. residual deviance with colony insects: binomial/probit = 15.578; binomial/logit = 16.649; quasibinomial: 16.649). Moreover, estimated LD50 and LD90 did not change between GLM models (e.g. LD50 with colony insects: binomial/probit = 0.0120 ng/insect; binomial/logit = 0.0120 ng/insect; quasibinomial = 0.0120 ng/insect). Estimated LD50 and LD90 were compared among *R. prolixus* origins using the ratio test suggested by Wheeler et al. [33] and performed with the *drc* R package [34]. If the confidence interval for the ratio of the LDs contains 1, no significant difference exists. The grade of resistance was determined as the ratio between LD50 in insects from palms (oil or native palms) and LD50 in insects from the susceptible colony [30].

Enzymatic activity was calculated as mass of product/mass of protein/time. Alfa-esterase activity was reported as nmol α-naphtol/mg protein/min, β-esterase activity as nmol β-naphtol/mg protein/min, and GST activity as nmol product/mg protein/min. MFO was not reported as enzymatic activity, but as µg of heme group/mg protein. Enzymatic activity was compared between *R. prolixus* origins by Kruskal-Wallis test (α = 0.05) and *a posteriori* pairwise contrasts by Wilcoxon tests (Bonferroni *P*-value adjusted method).

## Results

### Biological assays

**Fig. 2:**
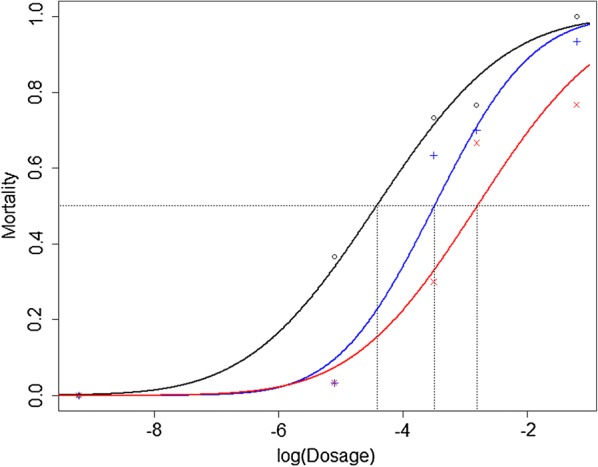
Response-dosage curves. Lines: probit logistic models. Points: mean mortality. *Key*: red, *R. prolixus* from oil palms; blue, *R. prolixus* from native palms; black, *R. prolixus* from a susceptible colony

The LD50 and LD90 values in *R. prolixus* from oil palms were significantly higher than those from the susceptible colony (LD50 Ratio = 5.055 ± 1.874 (estimate ± standard error), 95% CI: 1.381–8.729; LD90 Ratio = 4.797 ± 0.226, 95% CI: 4.354–5.239) (Table 1 and Fig. 2). In native palms, the LD90 value was significantly higher than in the susceptible colony (LD90 Ratio = 1.531 ± 0.157, 95% CI: 1.224–1.838), and LD50 was also higher but not significant (LD50 Ratio = 2.538 ± 1.389, 95% CI: − 0.184–5.261) (Table 1 and Fig. 2). The degree of resistance in oil palms was 5.042 and in native palms 2.542, the LD50 in *R. prolixus* from oil palms being 1.983 times higher than in native palms. Precisely, both lethal dosages were higher in oil palms than in native palms although LD50 was not significant (LD50 Ratio = 1.991 ± 0.517, 95% CI: 0.977–3.005; LD90 Ratio = 3.133 ± 0.120, 95% CI: 2.896–3.369). These results suggest the evolution of DM resistance in both oil and native palms. Nevertheless, it is important to note that the nymphs from the susceptible colony had a lower mean weight than those from native and oil palms (Table 1).

**Table 1:** Deltamethrin LD50 and LD90 in *R. prolixus* from each origin

Source	*n*^a^	Mass of each insect (mg)^b^	LD50 (ng DM/insect)^c^	LD90 (ng DM/insect)^c^
Susceptible	150	53.2 ± 12.3	0.0120 ± 0.0034	0.0966 ± 0.0382
Native palms	150	76.4 ± 8.24	0.0305 ± 0.0058	0.1480 ± 0.0497
Oil palms	150	79.5 ± 9.70	0.0605 ± 0.0142	0.4636 ± 0.2524

^a^Number of insects

^b^Mean ± standard deviation

^c^Regression estimate ± standard error

In assays with the commercial insecticide formulation, mean mortality was much lower in *R. prolixus* insects from oil palms than in those from the susceptible colony, and between palms, lower in oil palms than in native palms (Table 2), corroborating the higher insecticide resistance of *R. prolixus* from oil palms. In contrast to the assays with the pure DM, the nymphs from the susceptible colony did not have a lower mean weight than those from native and oil palms (Table 2).

**Table 2:** Mortality in *R. prolixus* from different sources caused by commercial deltamethrin

Source	*n*^a^	Mass of each insect (mg)^b^	Mean mortality (%)	
			Control	Insecticide^b^
Susceptible	40	80.2 ± 4.1	0	90.0 ± 10.0
Native palms	40	66.9 ± 3.9	0	43.3 ± 40.4
Oil palms	40	81.0 ± 4.2	0	10.0 ± 0

^a^Number of insects

^b^Mean ± standard deviation

### Biochemical assays

**Fig. 3:**
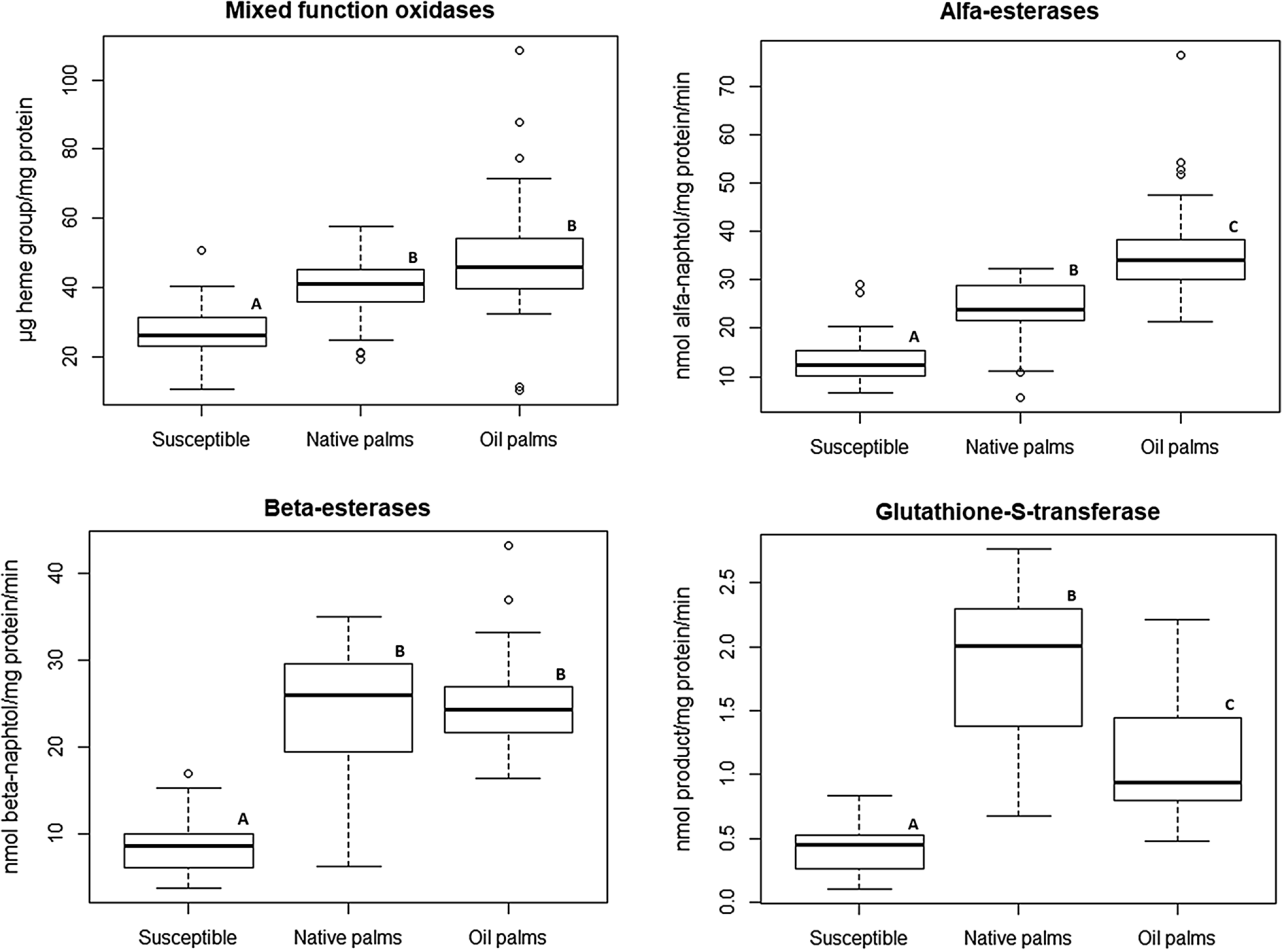
Activities of mixed function oxidases, alfa and beta esterases, and glutathione S-transferases in *R. prolixus* insects from the susceptible colony, native palms and oil palms. Pairwise comparisons (Wilcoxon test) were performed between *R. prolixus* of different origins, and those with the same letter (near to the boxplot) are not significantly different (α = 0.05)

Enzymatic activity differed significantly among *R. prolixus* from oil palms, native palms and the colony for all the detoxifying enzymes tested: MFO (Kruskal-Wallis test: *χ*^2^ = 34.421, *df* = 2, *P* < 0.0001), α-esterases (*χ*^2^ = 59.995, *df* = 2, *P* < 0.0001), β-esterases (*χ*^2^ = 51.121, *df* = 2, *P* < 0.0001) and GST (*χ*^2^ = 59.988, *df* = 2, *P* < 0.0001). MFO activity was higher in oil palms than in the colony (Wilcoxon rank sum test: *P* < 0.0001), but not different to native palms (*P* = 0.076) (Fig 3). Alfa-esterases activity was higher in oil palms than in native palms (*P* < 0.0001) and the colony (*P* < 0.0001) (Fig 3). Activity of β-esterases in oil palms was very similar to native palms, although it was still higher than in the susceptible colony (*P* < 0.0001) (Fig 3). Finally, GST activity in oil palms was higher than in the colony (*P* < 0.0001), but it was lower than in native palms (*P* = 0.0009) (Fig 3).

## Discussion

The present results show the evolution of deltamethrin resistance in insects collected from the field when compared to insects from the colony. Deltamethrin (DM) is a pyrethroid that acts on nerve membranes by delaying the closing of the activation gate for the sodium ion channel [35]. Pyrethroids are preferred for triatomine control in domiciles because they are applied at lower doses (cost-effective), have great acceptability both by householders and sprayers, are odorless, and leave no stains on the walls after application [3]. Pyrethroid resistance has been reported previously in triatomines; first evidence was reported in *R. prolixus* from Carabobo (Venezuela) and in *T. infestans* from Río Grande do Sul (Brazil) [36]. In our study, *R. prolixus* collected from oil palms would indicate resistance to DM pure and the commercial version. This possible resistance to DM in *R. prolixus* could be caused by continuous exposition to this insecticide. DM has been suggested for control of *Rh. palmarum* in oil palms [24], and this insecticide showed the lowest LD50 for *Rh. palmarum* in bioassays that evaluated several insecticides [24]. Control of *Rh. palmarum* is extremely important for oil palm plantations. Larvae tunnel into the crown and trunk, and the tissues around the growing point begin to decay and the palm may die [37]. Furthermore, there is an apparent association between *Rh. palmarum* and bud rot disease, a disease that has destroyed more than 70,000 ha of oil palm plantations in the western and central regions of Colombia [38].

An important consideration to include here is the possible role of the nymph weight on the previous results. In the biological assays with the pure DM, mean weight was greater in nymphs from native and oil palms (with lower mortality) than in those from the susceptible colony (with higher mortality) (Table 1). If insects only differed in individual weight, nymphs with higher weight would require a higher dose of insecticide to obtain the same toxicological result than nymphs with less weight. Differences in nymph weight as well as in insecticide susceptibility could explain the higher deltamethrin resistance found in nymphs from palms. However, this relationship between weight and mortality was not observed between native and oil palms, and in the commercial DM bioassays. Mean nymph weight in native and oil palms was very similar but their LD90s were significantly different (Table 1), and in commercial DM assays, nymph weight in the susceptible colony and oil palms were very similar but their mortalities were extremely different (Table 2). Therefore, we should not discard a possible effect of nymph weight on the obtained mortality values, but its impact has nonetheless shown no consistency among all the bioassays.

Enhanced metabolism could be occurring in *R. prolixus* from oil palms as a biochemical mechanism related to insecticide resistance. All detoxifying enzymes studied had higher activity in *R. prolixus* from oil palms than in the susceptible ones. Detoxifying enzymes could be acting over DM reducing the amount that affects the organism [3]. In *R. prolixus* populations from Venezuela and *T. infestans* from Brazil with resistance to deltamethrin, β-cyfluthrin and cypermethrin, monooxygenases activity was higher than in a susceptible lineage [36]. Moreover, esterases and monooxygenases have shown to be involved in pyrethroid resistance in *T. infestans* from Argentina [39–42].

Higher enzymatic activity in detoxifying enzymes may help *R. prolixus* insects to reduce their susceptibility to DM. Alfa- and beta-esterases may contribute to DM resistance by rapidly binding and slowly turning over the insecticide. Esterases apparently sequester insecticide rather than metabolize it [43]. MFOs are a large group of enzymes that play an important role in insects for the regulation of endogenous compounds and the metabolism of exogenous substances [43]. Elevated MFO activity has been associated with pyrethroid resistance [44]. GSTs are dimeric multifunctional enzymes that play a role in detoxification of a large range of xenobiotics including insecticides [45].

DM resistance was also observed in *R. prolixus* from native palms (pure and commercial version), but the degree of resistance was lower than in *R. prolixus* from oil palms. Additionally, all detoxifying enzymes studied, had higher activity in *R. prolixus* from native palms than in the susceptible ones.

Since native palms have not been sprayed with insecticides, possible resistance in *R. prolixus* from native palms could be related to insect migration between both palm types. Native and oil palms in the sampling site were very close (~30 m) and *Rhodnius* adults have demonstrated to fly much longer distances [46, 47]. Moreover, several vertebrate host species have been found in both riparian forest and oil palm plantations [11], and they could be circulating between these habitats transporting triatomines. Both palm types, native and oil palms have demonstrated suitable conditions for the sustainability of *R. prolixus* populations (e.g. host availability) [11, 26].

This scenario may be of concern for public health if resistant triatomines from oil palms colonize urban settings because traditional vector control may be inefficient. Triatomine migration from native palms toward houses has been demonstrated by morphometric and molecular studies [29, 48]. Triatomine migration can be triggered by the decrease of refuges and hosts by habitat degradation, human colonization of forested areas in low quality houses, and the attraction of bugs to artificial lights in houses at night [49–52].

Pest attacks in oil palm plantations may cause important economic losses for the oil palm cultivators. Colombia is currently the main oil palm producer in America. The expanding national and international biofuel market has stimulated much interest in biodiesel production in Colombia, especially given that the government has the ambitious goal of producing biodiesel, by replacing 20% of diesel with biofuel [53]. Nevertheless, triatomine presence in oil palm plantations should not be ignored, and it would be indicating a new important scenario of *T. cruzi* transmission [11].

Pest management in oil palm plantations must consider its impact over triatomines, and there is a need to consider a pest control strategy that also includes a triatomine vector control. We considered a complete strategy involving several procedures. First, the municipalities of Colombia with actual presence of triatomines in oil palm plantations must be identified. Entomological surveillance should be done in municipalities with both oil palm plantations and reported triatomine vectors (e.g. *Rhodnius prolixus*). Oil palm plantations and adjacent areas such as riparian forests should be monitored to determine triatomine presence. Here, the collaboration between oil palm cultivators and public health organizations is essential. Secondly, triatomine populations found in plantations should be toxicologically tested using biological or biochemical assays as those used in our study. The monitoring of triatomines should include population genetics studies identifying the presence and frequency of mutations related to insecticide resistance. Toxicological studies should include other pyrethroids also reported for triatomine control (e.g. cypermethrin and α-cyhalothrin) to determine if insecticide cross-resistance occurs as well. Thirdly, control strategies should be developed to impact successfully on both oil palm pests and palm-infesting triatomines. Insecticides and dosages used for oil palm pest control must also ensure triatomine control. Studies about the effect of current chemical pest control over triatomines should be performed. Then, insecticides, formulations or spraying frequencies should be modified to warrant the control of both pests and triatomines. Insecticide rotation can be considered also as a resistance management strategy. However, the rotation between insecticides such as organophosphates/carbamates and pyrethroids is not suggested based on toxic potency, toxicological risk, and quality of the formulations [3].

## Conclusions

*Rhodnius prolixus* collected from oil palms showed resistance to pure and commercial deltamethrin. This resistance may be caused by continuous exposition to the insecticide, which is suggested for the control of oil palm pests. Possible resistance mechanism in *R. prolixus* from oil palms are the increased activity of detoxifying enzymes, such as esterases and oxidases, which reduce the insecticide damage. Deltamethrin resistance was also observed in *R. prolixus* from native palms, but with a lower grade of resistance. Insecticide resistance in triatomines from oil palms may become a concern for public health if resistant triatomines migrate toward urban settings and hinder traditional chemical vector control. Pest management in oil palm plantations is extremely important for crop production; however, a pest control strategy that includes triatomine surveillance and toxicological monitoring should be considered. In the field of public health, these results are very important to redirect control strategies in triatomines, it is recommended to expand the studies to other areas to have more information.

## Data Availability

Data supporting the conclusions of this article are included within the article. The datasets generated and analyzed during the present study are available from the corresponding author upon reasonable request.

## Abbreviations

WHO: World Health Organization
LD50: lethal dosage 50
LD90: lethal dosage 90
RH: relative humidity
GST: glutathione S-transferases
MFO: mixed-function oxidases
